# Short and mid-term effects of modified release technique in rheumatic mitral valve repair

**DOI:** 10.1186/s13019-023-02254-w

**Published:** 2023-04-21

**Authors:** Hoshun Chong, Yaxuan Gao, Yunxing Xue, Xiyu Zhu, Jie Li, Junxia Wang, He Zhang, Dongjin Wang, Hailong Cao

**Affiliations:** 1 Department of Cardiothoracic Surgery, the Affiliated Drum Tower Hospital of Nanjing University Medical School, No.321 Zhongshan Road, Nanjing City, Jiangsu Province China; 2 Department of Ultrasound, The Affiliated Hospital of Nanjing University Medical School, Nanjing, China

**Keywords:** Mitral valve, Modified release, Rheumatic heart disease

## Abstract

**Objective:**

Repair or replacement remains debatable in rheumatic heart disease. To regain optimal mean transvalvular pressure gradients and end-diastolic peak flow velocity, the modified release technique combined peeling in the anterior leaflet and separated the shortened chordal. In the end, the short and mid-term outcomes of the modified release technique were evaluated.

**Methods:**

We retrospectively analyzed a series of 128 patients with rheumatic mitral stenosis, from January 2018 to July 2021 in our center. All patients undergoing mitral valve repair were using the modified release technique. The effect of mitral valve repair was evaluated by intraoperative transesophageal echocardiography and postoperative transthoracic echocardiography.

**Results:**

All the 128 patients successfully repaired the mitral valve. The intraoperative transesophageal echocardiography showed trivial or mild regurgitation. The aortic valve was repaired without obvious regurgitation in 12 cases, 5 cases received an aortic valve replacement, 89 cases underwent tricuspid annuloplasty. There were no blood transfusions in most patients, no deaths nor complications during peri-operation, also, no deaths and adverse events were observed during the follow-up period from 3 to 42 months. During the follow-up, 122 cases had no mitral valve regurgitation and 2 cases of moderate regurgitation, 4 cases of mild to moderate regurgitation. The mean peak flow velocity was 1.2 ± 0.3 m / s, no new-onset stenosis occurred.

**Conclusion:**

Modified release technique is safe and feasible. Its durability is acceptable in the short and mid-term, with no new-onset stenosis during the follow-up.

## Introduction

Rheumatic heart disease affects about 34 million people’s health in the world, causing 34,500 deaths per year and leading to about 30 thousand new-onset cases every year [1]. Rheumatic changes will cause mitral valve leaflet thickening, fibrosis, calcification, contracture, annulus enlargement, commissural, subvalvular chordae, and papillary muscle fusion, contracture, calcification, lengthy, or even rupture [2]. Considering the poor clinical outcomes and quality of life for rheumatic heart disease patients, valve surgery is considered the most effective treatment for rheumatic valvular disease [3, 4].

Considering its higher long-term survival rate and fewer complications, mitral valve repair surgery is considered the best method for treating valvular disease, especially degenerative mitral valve disease [5]. However, this opinion remains debatable in patients with rheumatic mitral valve disease [6]. Indeed, the majority of rheumatic valve disease patients, especially those with severe stenosis, received valve replacement instead of valve repair [7]. On the contrary, other studies showed that mitral valve repair in rheumatic valve disease patients had acceptable outcomes [8, 9]. So, whether or not should we choose mitral valve repair in rheumatic valve disease patients?

Here, we introduced a new modified release technique in dealing with rheumatic mitral valve lesions. The goal of this technique is to increase the maximum mitral valve effective orifice area and decrease mitral valve diastolic flow velocity. We reported the mid & long-term follow-up results of the modified release technique in 128 patients and compared them with the results of mitral valve replacement.

## Methods

### Patient enrollment

From January 2018 to July 2021, patients with Type III rheumatic mitral valve stenosis [10]who underwent heart surgery by Dr. Cao in Nanjing Drum Tower Hospital were selected, our team performed 264 mitral valve repair and 285 mitral valve replacement in these patients. Concomitant atrial radio-frequency ablation, tricuspid valve repair, aortic valve repair, or replacement was not excluded. 128 patients underwent modified release technique and 128 matched patients underwent mitral valve replacement based on age and gender. Transesophageal echocardiography assessment was routinely performed under anesthesia before and after the operation. Transthoracic echocardiography was performed to evaluate postoperative outcomes during follow-up. This study was approved by the institutional review board of Nanjing Drum Tower Hospital (Number: 2021-604-01). Informed consent was exempt because of the retrospective nature of the study.

### Surgical technique

**Fig. 1:**
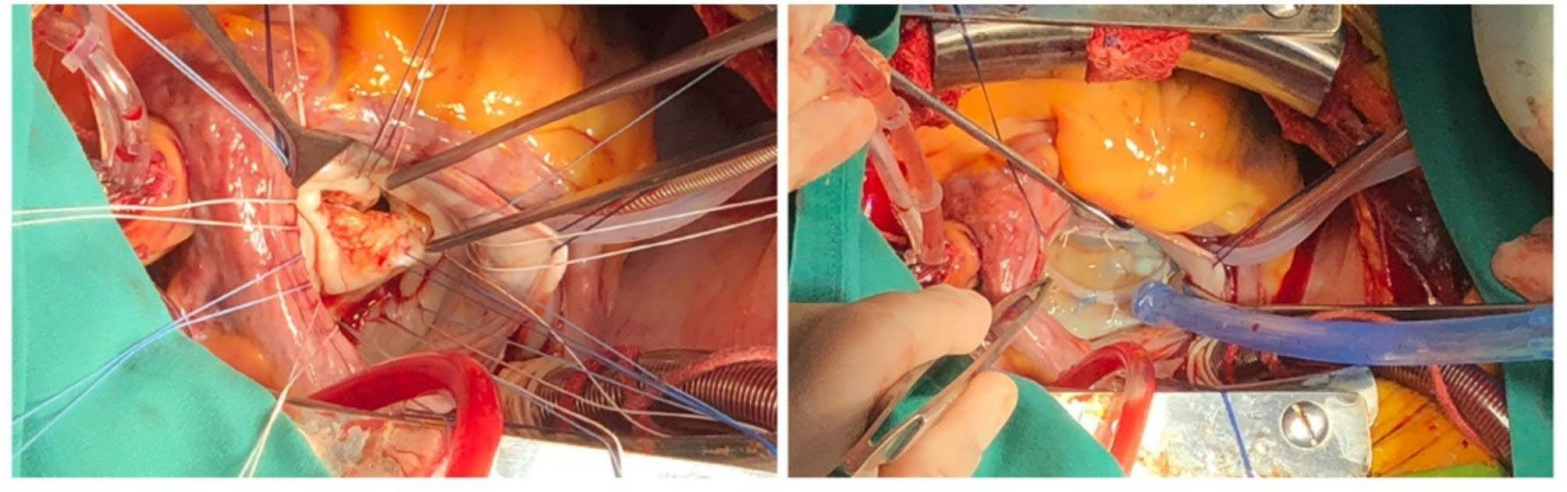
Modified release technique for rheumatic mitral stenosis valvuloplasty. (Left) Leaflet thickening, calcification, and contracture of the mitral valve before MVP. (Right) Physiological bulging of the leaflet, adequate coaptation area and no obvious reflux in water test after MVP.

**Fig. 2:**
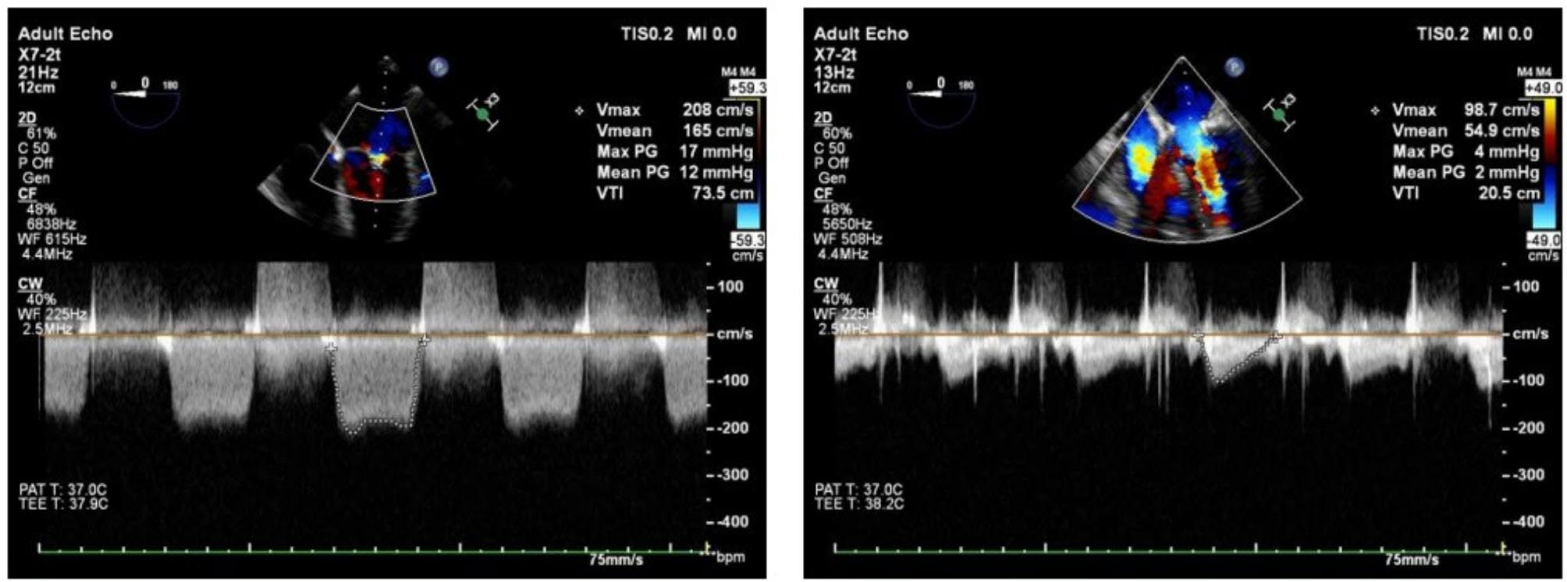
Intraoperative transesophageal ultrasound. (Left) Pre-MVP, end-diastolic peak flow velocity is 2 m/s, and Transvalvular pressure gradients are 17 mmHg. (Right) Post-MVP, End diastolic peak flow velocity 1 m/s, and Transvalvular pressure gradient is reduced to 4 mmHg

Media-sternotomy or right infra-axillary vertical thoracotomy was the surgical approach for all patients, our standard operating procedure for right infra-axillary vertical thoracotomy was published [11]. After systemic heparinization, cardiopulmonary bypass is established through ascending aorta and superior/inferior vena cava. Then the mitral valve was exposed through the right atrium opening and trans-septal approach. The posterior mitral annuloplasty line was sutured to expose the anterior and posterior commissure. A 15-blade was used to remove the commissural fibrous plaques, thin the commissure, then we performed commissurotomy to the commissural body according to the actual commissural border. Then we evaluated the subvalvular apparatus using a nerve hook. We then used a 15-blade to separate the shortened chordal (preserve the paracommissural chordal) and perform papillary muscle dissection for fused papillary muscle. When the coaptation area and chordal were severely thickened and fused we performed chordal fenestration under the coaptation area. The peeling technique was performed at the anterior leaflet only. We performed the first water test before inserting the ring. Then after the ring was inserted, we perform a second water test to assess the quality of the repair (Fig. 1). Transesophageal echocardiography assessment was routinely performed under anesthesia before and after operation to evaluate the mean transvalvular pressure gradients and end-diastolic peak flow velocity (Fig. 2). This method increased the repair possibility to up to approx. 60% in type III patients.

### Statistical analysis

Statistical analysis was executed with SPSS 22.0 package (SPSS Inc., Chicago, IL, USA). For the comparison between the two groups, Student’s *t*–test (normally distributed) or Mann–Whitney test (non-normally distributed) was used for continuous variables, and the *c*^*2*^ test was used for categorical variables. A *p*-value < 0.05 was defined as statistically significant.

## Results

### Demographic data

Patients were divided into the repair group and the replacement group as mentioned in Method. The follow-up period was 3–42 months. The mean ages were 52.7 ± 10.8 years and 52.9 ± 10.4 years, respectively. The female/male ratio was the same in both groups. The pre-operative effective orifice area was 1.2 ± 0.2 cm^2^ in the repair group and 1.2 ± 0.3 cm^2^ in the replacement group. Although in the replacement group there was a higher prevalence of NYHA III/IV and left atrial appendage thrombus, these differences had not reached statistical significance (Table 1).

**Table 1:** The baseline demographics and clinical variables in rheumatic mitral valve repair and replacement

Clinical Variables	MVP(n = 128)	MVR(n = 128)	P-value
Age(y)	52.7 ± 10.8	52.9 ± 10.4	0.89
Gender(Male/Female) (n)	51/77	51/77	1.00
BMI (kg/m2)	23.7 ± 0.3	24.1 ± 0.3	0.27
BSA (m2)	1.66 ± 0.01	1.69 ± 0.01	0.32
NYHA classification (I/II/III/IV)(n)	4/78/42/4	6/62/52/8	0.201
Preoperative			
Mitral valve area(cm^2^)	1.2 ± 0.2	1.2 ± 0.3	0.97
Left atrial appendage thrombi(n)	14	20	0.269
LAD (mm)	50.5 ± 0.8	51.3 ± 0.7	0.44
LVD(mm)	52.4 ± 0.7	53.0 ± 0.7	0.50
Pulmonary Pressure	35.6 ± 0.7	35.5 ± 0.6	0.89
Operative and perioperative variables			
Concomitant procedure			
RFCA(n)	46	89	< 0.01
Aortic Valve surgery(n)	17	27	0.10
TVP(n)	89	112	< 0.01
right infra-axillary vertical thoracotomy(n)	27	11	< 0.01
CPB time(min)	105 ± 15	125 ± 28	< 0.01
X-clamp time(min)	70 ± 16	85 ± 29	< 0.01
Mechanical Ventilation(h)	7.2 ± 2.7	9.3 ± 3.7	< 0.01
Post-operative discharge(d)	7.6 ± 2.1	8.9 ± 2.6	0.03
End diastolic peak flow velocity(m/s)	1.2 ± 0.3	1.6 ± 0.4	< 0.01
Transvalvular pressure gradients (mmHg)	2.7 ± 1.1	3.8 ± 1.2	0.01

### Operative data

Patients in the replacement group underwent more concomitant atrial radio-frequency ablation, tricuspid valve repair, aortic valve repair, or replacement than the repair group. 27 patients underwent right infra-axillary vertical thoracotomy instead of media-sternotomy in the repair group while only 11 patients underwent right infra-axillary vertical thoracotomy in the replacement group. The overall CPB and X-clamp time were all significantly shorter in the repair group (105 ± 15 min vs. 125 ± 28 min and 70 ± 16 min vs. 85 ± 29 min, respectively; P < 0.01) (Table 1).

### Perioperative and follow-up outcomes

All patients in the repair group successfully underwent modified release technique without failure repair attempt and underwent a replacement procedure. Post-plasty TEE showed mitral valve regurgitation no more than mild. 46 atrial fibrillation patients, 37 recover sinus rhythm after surgical ablation, 9 recover sinus rhythm through electric cardioversion. 12 patients underwent concomitant aortic valve repair, 5 aortic valve replacement, and 89 tricuspid valve repairs. All patients implanted 30-34 mm Carpentier-Edwards Physio I/II annuloplasty ring or Medtronic CG Future annuloplasty ring. The mean transvalvular pressure gradients were 2.7 ± 1.1 mmHg and end-diastolic peak flow velocity was 1.2 ± 0.3 m/s in the repair group, these results were better compared with 3.8 ± 1.2 mmHg (P = 0.01) and 1.6 ± 0.4 m/s (P < 0.01) in replacement group. Post-operative mechanical ventilation and hospital stay were significantly shorter in the repair group (7.2 ± 2.7 h vs. 9.3 ± 3.7 h, P < 0.01 and 7.6 ± 2.1 d vs. 8.9 ± 2.6 d, P = 0.03, respectively). During the 3–42 months follow-up, no deaths or cardiovascular/valvular plasty related adverse events occurred. ECG showed all patients remain sinus rhythm. At the recent clinic visit, TTE of patients undergoing modified release showed 81 patients with trivial regurgitation, 41 patients with mild regurgitation, 4 patients with mild-moderate regurgitation, and 2 with moderate regurgitation. None showed new onset mitral valve stenosis.

## Discussion

The average age of patients with rheumatic mitral valve disease in China is 40–55 years old [12]. The first choice for these patients’ treatment in most centers is mitral valve replacement. However, the risk of anticoagulation/bleeding of mechanical valves and early bioprosthetic valves deterioration in young patients [13] affects the patients’ long-term survival and quality of life. Thus, a feasible and effective mitral valve repair for young rheumatic valve patients is required.

Asian countries such as Thailand, India, Vietnam, and Malaysia have shown good therapeutic effects in rheumatic mitral valve repair [14]. They use a pericardial patch for anterior or posterior mitral valve extension to repair the rheumatic mitral valve [15] and the Peeling technique for both leaflets [16]. However, the pathology of the Chinese rheumatic heart disease population is different. Patients in Asian countries such as Thailand, India, Vietnam, and Malaysia are younger, and the main population is adolescents. The valve is usually not yet mature. In the meanwhile, Chinese patients are normally in their 50s/60s [17]. The size of the leaflet is normal and the coaptation area is sufficient therefore does not require widening. Furthermore, after a thorough peeling in both leaflets, in order to acquire better leaflet morphology and coaptation height, require a down-sized annulus ring. For Chinese rheumatic patients, we do not recommend using a small ring, due to the normal size of the leaflet and adequate coaptation height. A down-sized ring will bring no benefit to mean transvalvular pressure gradients and end-diastolic peak flow velocity. So as to remain an actual-sized annulus, additional leaflet extension is necessary. All procedures performed simultaneously are complicated and highly increase the risk of repair failure. We only perform the Peeling technique in the anterior leaflet to remove fibrous plaque that affects coaptation. The posterior leaflet is thickened and fixed and does not play a key role in the opening of the mitral valve.

Another important aspect of the mitral valve structure is the subvalvular apparatus. The Score procedure introduced by Meng and colleagues is a simple operation and with reliable effects and easy to promote [18]. This procedure had shown many encouraging clinical outcomes in severe rheumatic mitral stenosis [19, 20]. On the basis of this procedure, we further focus on subvalvular apparatus. Modified release technique separates the shortened chordal (preserve the paracommissural chordal), performs papillary muscle dissection for fused papillary muscle and chordal fenestration under the coaptation area. This technique ensures the maintenance of mitral valve structure to ensure the best preload state of the left ventricle. On the other hand, leaflet and subvalvular apparatus were removed during valve replacement, causing left heart function was also lost. This is one of the important reasons why the long-term survival rate after valve replacement is less satisfactory [9].

In recent years, whether mitral valve repair or replacement is better in rheumatic heart patients remains debatable [21, 22]. There might be a few possible reasons: [1] rheumatic mitral valve disease has more complicated pathological changes in the valve, annulus, and subvalvular apparatus; [2] surgeon skills and experience matter in valve repair; [3] the potential failure of valve repair and tendency of valve replacement in mixed rheumatic lesion; [4] durability of mitral valve repair in rheumatic patients. Our modified release technique, according to the pathological change of adult rheumatic valve patients, peeling in the anterior leaflet, separating the shortened chordal and chordal fenestration are feasible and received acceptable mid & long-term results. Echo showed lower mean transvalvular pressure gradients and end-diastolic peak flow velocity. These changes are important in relieving patients valve-related symptoms and the durability of valve repair.

## Limitations

This study has several limitations. First, this is a retrospective study from a single institution. Second, patients’ selection bias is inevitable since patients’ treatment was sometimes selected based on patients’ conditions. As a consequence, we were not able to evaluate the outcome of different surgical techniques as we did not perform randomized surgical choices. Third, we did not compare between different repair techniques, of which, further study is needed.

## Conclusions

In conclusion, the modified release technique is safe and feasible. Its durability is acceptable in the long-term, with no new-onset stenosis during follow-up. The echo shows that this technique lowers mean transvalvular pressure gradients and end-diastolic peak flow velocity. Therefore, the modified release technique is a useful repair technique in surgeons’, especially young surgeons’ toolboxes.

## Data Availability

The datasets generated and analyzed during the current study are available from the corresponding author on reasonable request.

## Abbreviations

CPB: cardiopulmonary bypass
TEE: trans esophageal echocardiography
ECG: electrocardiogram
NYHA: New York Heart Association
RFCA: radiofrequency current catheter ablation
TVP: tricuspid valve plasty
